# A cross-sectional study on burnout and its individual and environmental correlates among hepatological surgery nurses in Hunan Province, China

**DOI:** 10.1371/journal.pone.0283373

**Published:** 2023-03-23

**Authors:** Honghui Zhang, Yuting Xiao, Ting Dai, Qian Li, Ling Huang, Xiu Huang, Dan Liu, Yu Yu, Jia Guo

**Affiliations:** 1 Department of Hepatobiliary Diseases, Hunan Provincial People’s Hospital: The First-Affiliated Hospital of Hunan Normal University, Hunan, China; 2 Department of Nursing, Hunan Provincial People’s Hospital: The First-Affiliated Hospital of Hunan Normal University, Hunan, China; 3 Department of Psychiatry, Division of Prevention and Community Research, Yale School of Medicine, New Haven, Connecticut, United States of America; 4 Department of Clinical Nursing, Xiangya School of Nursing, Central South University, Hunan, China; Lithuanian University of Health Sciences, LITHUANIA

## Abstract

**Background:**

Burnout is a widespread occupational phenomenon among nurses with significant adverse outcomes for nurses, patients, and society. It is thus important and urgent to understand burnout and its risk factors to guide interventions. This study aimed to examine the level of burnout and explore its individual and environmental correlates.

**Methods:**

This cross-sectional study was conducted in Hunan, China. A total of 623 hepatological surgery nurses completed an online survey (response rate: 72.78%). Burnout was measured using the standard Maslach Burnout Inventory (MBI). Information on individual factors and environmental factors was collected by self-designed questionnaires.

**Results:**

The scores of emotional exhaustion, depersonalization, and personal achievement in nurse burnout were 30 (26–34), 11 (8–14), and 23 (20–26) respectively. The prevalence of high burnout ranged from 52.81% for emotional exhaustion to 90.37% for decreased personal achievement. The three dimensions of burnout shared common correlates such as self-rated physical health and working environment, while also having additional unique correlates such as overwork, satisfaction with income, and age.

**Conclusion:**

Hepatological surgery nurses in Hunan Province are suffering from high levels of burnout, which requires public attention and urgent interventions. Improvement of the physical health and working environment of nurses may be the most beneficial intervention measures to tackle various dimensions of burnout, while other targeted measures are also needed for each specific dimension.

## Introduction

Burnout refers to a psychological syndrome in response to chronic and enduring emotional and interpersonal stress on the job [1,2]. The World Health Organization (WHO) has recently declared burnout as an “occupational phenomenon that has not been successfully managed” in the International Classification of Diseases 11th revision (ICD-11) [3]. Burnout is conceptualized as a tripartite construct characterized by three domains: emotional exhaustion (EE), depersonalization (DP), and a diminished sense of personal accomplishment (PA) [4]. EE, as the central component and first step of burnout, refers to the emotional overextension and depletion resulting from work stress. DP, as a consequent interpersonal aspect of burnout, refers to callous, negative, pessimistic, or detached responses toward fellow workers and clients. Reduced PA, as the self-evaluation aspect of burnout, refers to the feelings of incompetence and dissatisfaction with one’s work productivity and achievement.

Burnout is a widespread occupational phenomenon that runs rampant among healthcare workers, especially nurses who acted as frontline caregivers in the provision of holistic patient care [5,6]. Excessive workloads, irregular working hours, rotating shifts, and understaffing all contribute to unrelenting physical and psychological stress among nurses and leave them particularly susceptible to burnout [7,8]. Globally, at least one in ten nurses has experienced burnout, according to a recent meta-analysis covering nurses across multiple specialties in 49 countries [9]. Nationally, the prevalence of burnout was reported to be as high as 50% in the United States [6], 49% in China [10], 41% in Thailand [11], and 60% in South Korea and Japan [12]. The already high level of burnout among nurses worldwide is further amplified by the ever-growing stress due to the coronavirus disease 2019 (COVID-19) pandemic, placing nurses at an even higher risk of burnout.

Burnout has negative consequences on both the physical and mental health of nurses, leading to physical discomforts such as fatigue, headache, insomnia, appetite changes, chest pain, frequent colds, gastrointestinal distress, and heart disease, as well as mental dysfunction such as reduced concentration and memory, irritability, depression, anxiety, and even mortality [13,14]. Apart from endangering the health and well-being of nurses themselves, burnout also exerted adverse effects on the patients through nurse underperformance and poor nurse-patient relationships [15,16]. Abundant evidence has consistently shown that burnout nurses were more likely to miss essential parts of patient care such as medication delivery, communication, and surveillance [17]. Nurse burnout has been demonstrated to be associated with higher rates of medical errors, patient mortality, failure to rescue, prolonged length of stay, and compromised patient safety [18,19]. In addition, nurse burnout also brings about negative organizational and societal implications, leading to higher rates of job withdrawal such as absenteeism, presentism, intention to leave, and actual turnover [18,20]. A recent national study in the US showed that 31.5% of the nurses who left their job reported burnout as a major reason [21], and there will be a global deficit of nearly 7.6 million nurses by 2030 as estimated by the WHO [22].

Nurse burnout, as an important and serious public health issue, requires worldwide attention and action. This is reflected in a recent call for expansion from the “Triple Aim” [23] which focuses on improving patient experience, improving population health, and reducing healthcare costs to the “Quadruple Aim” which adds reducing burnout [24], indicating the importance and urgency to address burnout. Due to the physical, psychological, and social toll burnout takes on nurses, it is essential and urgent to understand the risk factors that lead to burnout to guide further targeted interventions. The past scholarship has invested much effort in exploring the antecedents to nurse burnout, which may be generally classified into individual factors and environmental factors. Some common individual factors have been identified to be associated with burnout, including age, gender, education, marital status, income, number of kids, working hours per week, physical health, etc. In general, being male [25,26], having higher education [25,27], having a higher income [25,28], being unmarried [29,30], having no kids [29,31], longer working hours per week [21,25,32], and poor physical health [25,32] are associated with a higher level of burnout. For environmental factors, substantial evidence has documented that poor and stressful work environment, such as poor staffing ratios, increased workloads, lack of professional autonomy, lack of leadership support, and lack of colleague communication and collaboration, is associated with nurse burnout [12,19,21,29,33,34].

Although the literature is replete with research on nurse burnout, most studies did not distinguish nurses from different departments or focused on nurses from certain departments such as emergency, oncology, pediatric, primary, anesthesia, and hemodialysis. To our knowledge, little is known about nurse burnout in the hepatological surgery department with predominantly liver cancer patients, especially in China which used to be labeled “the leader in liver diseases” [35]. Liver diseases are one of the leading causes of morbidity and mortality in China [36]. It is estimated that over 20% of the Chinese population is affected by liver diseases, including hepatitis B virus (HBV) and hepatitis C virus (HCV) infections, liver cirrhosis, liver cancer, non-alcoholic fatty liver disease (NAFLD), and alcohol-related liver disease (ALD) [35]. Based on recent epidemiological data, HBV, a leading cause of liver cancer, affects 90 million (6.52%) people in China, as compared to 2.2 million (0.71%) in the US [35]. According to a 2018 report by the International Agency for Research on Cancer, China has the 9th highest rate of liver cancer and the largest number of liver cancer in the world [37]. The high prevalence and huge disease burden of liver diseases in China pose significant challenges to the healthcare workers in the hepatological surgery department, especially nurses who are on the frontline to deal with patients directly [38]. It is thus crucial to understand the level of nurse burnout in the hepatological surgery department and identify risk factors to guide future intervention and improvement plans. The current study was conducted to examine the level of burnout that includes three dimensions (EE, DP, and PA), as well as explore two types of risk factors (individual and environmental), among a provincial sample of hepatological surgery department nurses.

## Methods

### Participants and procedure

This cross-sectional study was carried out in all 79 tertiary hospitals that have set up independent hepatological surgery departments in Hunan Province, China between June 2021 to July 2021. Since this was a provincial-level epidemiological study, we aimed to include as many nurses as possible to get an unbiased representative sample that included all eligible nurses for this study and to get a comprehensive understanding of the current level of nurse burnout. To realize this goal, we retrieved the name list of all registered nurses in all hepatological surgery departments in Hunan Province through the Hepatobiliary Health Education Chair of Hunan Province and approached each nurse for the study enrollment. The minimal sample size was calculated according to the form for cross-sectional study: n = z^2^ P(1 − P) / E^2^, where P (the prevalence of burnout) was estimated at a minimum of 40% based on past studies, Z was set as 1.96 at a confidence interval of 95%, an allowable error was set as 5%. Considering a rejection or lost-to-follow-up rate of 20%, we got a final minimal sample size of 443. The inclusion criteria of the subjects were as follows: (a) full-time registered nurses with effective professional qualification certificates; (b) specialized hepatological nurses; (c) aged ≥ 18. The exclusion criteria were as follows: (a) nurses who rotated, visited, and/or studied in the hepatological surgery department; (b) nurses who were on leave due to sickness, maternity, or other reasons during the survey; (c) nurses who were unable to fill in the online questionnaire due to physical or mental illness. A list of all eligible nurses was achieved from each hepatological surgery department in each hospital after the official permission was granted. The eligible nurses were invited to participate in an online survey through Sojum [39]—a most popular professional online survey tool in China that provides multiple services including questionnaire design and distribution, data collection, and analysis. All nurses were fully informed of the study’s purpose and procedure. Since the study was conducted online instead of face-to-face, all participants provided their written informed consent electronically through Sojum before filling in the online questionnaire. In order to protect participant privacy, each questionnaire was de-identified and authors had no access to any information that could identify individual participants during or after data collection.

In total, 856 nurses from the hepatological surgery departments of the 47 hospitals were eligible for the study and invited to participate in the online survey. After excluding 146 nurses who refused to respond, 38 nurses with obvious logical mistakes in their answers, and 49 nurses with a too-short response time in filling in the questionnaire (3 SD below the average response time), our final effective sample was 623 (response rate:72.78%), which satisfies the minimal sample size requirement of 443. Each item of the online questionnaire was required to be filled in before submission and as a result, there is no missing data in the final dataset. Ethics approval was obtained from the Institutional review board (IRB) of the Hunan Provincial People’s Hospital (No.: Ky-2021-65).

### Measures

The questionnaire for this study included the following three parts: (a) individual factors that include both socio-demographic information and work-related characteristics; (b) environmental factors that include hospital-level, department-level, colleague-level, and patient-level factors; and (c) the outcome variable of burnout that includes the following three dimensions: EE, DP, and PA.

#### Individual factors

Individual factors included socio-demographic and work-related characteristics. Socio-demographic data of the nurses included age, gender (male/female), marital status (married/not married), educational level (Technique school and below /college and above), number of kids (0/1/≥2), satisfaction with income (rated on a five-point Likert scale ranging from 1 = extremely unsatisfied to 5 = extremely satisfied, which was further categorized as not satisfied = 1,2/ neutral = 3/ satisfied = 4,5), and self-rated physical health (rated on a five-point Likert scale ranging from 1 = extremely bad to 5 = extremely good, which was further categorized as poor = 1,2/neutral = 3/good = 4,5). Work-related characteristics included duration of nursing (<10/≥10 years), employment types (permanent/contract nurse), professional rank (primary/junior/senior), job title (nurse/nursing team leader/head nurse), working hours per week (<40/≥40 hours), night shift (no/yes), work overtime (no/yes), additional work assignment (no/yes).

#### Environmental factors

The working environment was evaluated with a self-designed 4-item working environment scale that was specifically designed for this study. The scale asked about the importance and respect participants received from the following four sources of work: the hospital, the department, colleagues, and patients. For the hospital and department level, the participants were asked if the hospital/department attached enough importance to the development of nursing in the hepatological surgery department. For the colleague level, participants were asked if they had harmonious relationships with their colleagues. For the patient level, participants were asked if they were respected by their patients. Each item was rated on a five-point Likert scale ranging from ‘1’ (extremely no) to ‘5’ (extremely yes). The total score of the working environment scale ranges from 4 to 20, with a higher score indicating a better working environment for the nurses. In this study, the mean value of 16 was used as a cutoff to distinguish those with a poor and good working environment. The working environment scale showed acceptable internal consistency with a Cronbach’s alpha coefficient of 0.78.

#### Burnout

Burnout was measured by the Maslach Burnout Inventory (MBI), which is the most widely used standard scale for workplace burnout all over the world [40]. The MBI includes 22 items under three dimensions: emotional exhaustion (EE) characterized by extreme tiredness due to excess efforts (9 items), depersonalization (DP) characterized by negative attitudes in interpersonal relationships (5 items), and personal accomplishment (PA) characterized by negative self-assessment (8 items) [40]. Each item is rated on a seven-point Likert scale ranging from ‘0’ (never) to ‘6’ (every day). Sample items include “I feel emotionally drained from my work,” “I don’t really care what happens to some recipients,” and “I deal very effectively with the problems of my recipients.” The total score of each subscale ranges from 0–54 for the EE, 0–30 for the DP, and 0–48 for the PA, with scores of ≥27 on EE, ≥10 on DP, or ≤33 on PA representing high burnout for the associated dimension [40]. In addition, to get a combined score for the MBI, we reverse-coded each item of the PA to get a transformed subscale—decreased personal accomplishment (DPA) [26]. The total score of MBI was calculated by summing up the scores of EE, DP, and DPA and ranges from 0–132, with higher scores indicating higher levels of burnout. The Chinese version of MBI was first validated by You et al. and has been widely used in the Chinese population [41]. In the current study, the MBI showed good internal consistency with a Cronbach’s alpha coefficient of 0.91.

#### Data analysis

All data were analyzed using STATA® version 13. Sample characteristics were examined using descriptive statistics, with continuous variables represented by means ± standard deviation, and categorical variables by numbers and percentages in each category. Comparisons of burnout scores including EE, DP, and DPA by individual and environmental characteristics were conducted by t‐test or ANOVA test as appropriate. Correlates of burnout and its three dimensions were explored by multivariate linear stepwise regression analysis with backward selection.

## Results

### Sample characteristics

Table 1 provides a detailed summary of the sample characteristics that include individual factors (socio-demographic and work-related factors), environment factors (hospital, department, colleague, and patient-level), as well as the primary outcome burnout score (EE, DP, DPA). For socio-demographic characteristics, the nurse profile was consistent with a 30-year-old married female with a college education and with kids. Regarding work-related characteristics, most nurses were in their junior nursing career with primary titles and were generally unsatisfied with their income. They also had long working hours, night shifts, additional work assignments, and overtime work. As for the working environment, only half reported a good working environment. For job burnout, the nurses reported an alarmingly high level of burnout, with the prevalence of high EE, DP, and PA being 52.81%, 83.79%, and 90.37%, respectively.

**Table 1:** Individual characteristics of the study population (N = 623).

Variables		Variables		Variables	
	M ± SD /N (%)		M ± SD /N (%)		M ± SD /N (%)
Individual factors					
Socio-demographical characteristics		Work-related characteristics		Environmental factors	
Age	30.88±6.14	Years of Nursing	9.48±6.52	Work environment	15.57±2.51
<30	283 (45.43)	<10	367 (58.91)	Poor	293 (47.03)
30–39	288 (46.23)	≥10	256 (41.09)	Good	330 (52.97)
≥40	52 (8.35)	Employment types		**Burnout Total**	66.30±20.65
Gender		Contract nurse	353 (56.66)	Emotional exhaustion	27.63±10.06
Male	8 (1.28)	Permanent nurse	270 (43.34)	No	294 (47.19)
Female	615 (98.72)	Professional ranks		Yes	329 (52.81)
Marital status		Primary nurse	317 (50.88)	Depersonalization	14.01±5.15
Not married	206 (33.07)	Junior nurse	264 (42.38)	No	101 (16.21)
Married	417 (66.93)	Senior nurse	42 (6.74)	Yes	522 (83.79)
Education		Job title		Personal accomplishment	23.34±7.28
Technique and below	113 (18.14)	General nurse	463 (74.32)	Decreased personal accomplishment	24.66±7.28
College and above	510 (81.86)	Nursing team leader	110 (17.66)	No	60 (9.63)
Number of kids		Head nurse	50 (8.03)	Yes	563 (90.37)
0	245 (39.33)	Working hours/ week	41.51±13.00		
1	227 (36.44)	<40	130 (20.87)		
≥2	151 (24.24)	≥40	493 (79.13)		
Satisfaction with income	2.91±0.86	Night shift			
Not satisfied	185 (29.70)	No	163 (26.16)		
Neutral	297 (47.67)	Yes	460 (73.84)		
Satisfied	141 (22.63)	Work overtime			
Self-rated health	3.28±0.80	No	309 (49.60)		
Poor	89 (14.29)	Yes	314 (50.40)		
Neutral	302 (48.48)	Additional work assignment			
Good	232 (37.24)	No	257 (41.25)		
		Yes	366 (58.75)		

### Burnout scores by individual and environmental factors

Table 2 presents a comparison of various burnout scores (MBI, EE, DP, DPA) by individual and environmental factors. For both the total MBI scale and its three subscales, nurses who were satisfied with income, in good self-rated health, and good working environment showed significantly lower burnout scores than their counterparts. Nurses who worked ≥40 hours per week, worked overtime, and had additional work assignments reported significantly higher burnout scores than their counterparts. In addition, females reported higher burnout scores than males, reflected in the total MBI scale and the EE subscale. Contract nurses and nurses with nightshifts reported higher burnout scores than their counterparts, reflected in the EE subscale only.

**Table 2:** Comparisons of burnout scores by various individual and environmental characteristics.

Variables	Burnout total score	P	Emotional exhaustion	P	Depersonalization	P	Decreased personal achievement	P
Individual factors								
Socio-demographics								
Age								
<30	65.30 ± 21.48	0.151	27.43 ±10.26	0.070	13.89±5.52	0.521	23.98±7.41	0.079
30–39	67.89±20.20		28.31±9.90		14.23± 4.93		25.35±7.28	
≥40	62.90± 17.92		24.88± 9.53		13.44±4.13		24.58±6.26	
Gender								
Male	50.38± 7.31	**0.028**	19.75± 4.37	**0.026**	10.63±4.03	0.061	20.00±3.30	0.068
Female	66.51 ±20.69		27.73± 10.07		14.05±5.15		24.72±7.30	
Marital status								
Not married	66.08±21.30	0.855	27.75± 10.42	0.826	14.09±5.60	0.779	24.24± 7.14	0.306
Married	66.41±20.35		27.56± 9.89		13.97±4.91		24.87±7.34	
Education								
Technique and below	63.99±22.38	0.19	26.96± 10.50	0.434	13.58±5.41	0.332	23.45±8.22	0.0504
College and above	66.81±20.23		27.77± 9.97		14.10±5.09		24.93±7.03	
Number of kids								
0	66.22±21.63	0.837	27.84± 10.49	0.525	14.11±5.59	0.916	24.28±7.33	0.540
1	65.83±19.51		27.04± 9.61		13.99± 4.80		24.80±6.99	
≥2	67.12±20.79		28.16± 10.03		13.89±4.91		25.07±7.63	
Satisfaction with income								
Not satisfied	72.81±21.00	**<0.001**	31.39±9.80	**<0.001**	15.81±5.41	**<0.001**	25.61±7.56	**0.030**
Neutral	65.95±20.42		27.54± 9.67		13.76±5.02		24.64±7.39	
Satisfied	58.50±17.75		22.87± 9.18		12.17±4.25		23.46±6.48	
Self-rated health								
Poor	76.19± 18.30	**<0.001**	32.98± 8.29	**<0.001**	16.30±5.01	**<0.001**	26.91±6.79	**0.001**
Neutral	68.78± 20.38		29.42±9.62		14.59±5.25		24.77±7.27	
Good	59.28± 19.60		23.23±9.55		12.38±4.55		23.66±7.29	
**Work-related characteristics**								
Years of nursing								
<10	66.54±21.03	0.725	27.88± 10.12	0.451	14.10±5.34	0.586	24.56±7.30	0.669
≥10	65.95±20.12		27.26± 9.98		13.88±4.85		24.81±7.26	
Employment types								
Contract nurse	67.42±21.44	0.122	28.54± 10.25	**0.009**	14.28±5.45	0.137	24.60±7.56	0.816
Permanent nurse	64.84±19.51		26.44± 9.70		13.66±4.71		24.74±6.91	
Professional ranks								
Primary nurse	66.02± 21.60	0.942	27.84±10.24	0.733	13.93± 5.57	0.904	24.25±7.46	0.243
Junior nurse	66.61± 19.86		27.54± 9.95		14.12±4.73		24.95±7.17	
Senior nurse	66.45± 18.48		26.57±9.52		13.90± 4.32		25.98±6.41	
Job title								
General nurse	66.31±21.41	0.463	27.86±10.27	0.087	14.07±5.35	0.475	24.37±7.49	0.241
Nursing team leader	67.64±18.63		28.00±9.49		14.13±4.66		25.51±6.73	
Head nurse	63.26±17.50		24.62±8.90		13.16±4.10		25.48±6.28	
Working hours/week								
<40	61.32±20.99	**0.002**	25.05± 9.86	**0.001**	12.93±5.24	**0.007**	23.35±7.50	**0.020**
≥40	67.61±20.38		28.31±10.01		14.29±5.09		25.01±7.19	
Night shift								
No	64.47±20.77	0.188	25.99±10.13	**0.016**	13.67±4.93	0.3341	24.80± 7.52	0.784
Yes	66.95± 20.59		28.20±9.98		14.13±5.22		24.62±7.20	
Work overtime								
No	62.86±20.92	**<0.001**	25.47±10.20	**<0.001**	13.34±5.03	**0.001**	24.05±7.44	**0.038**
Yes	69.68±19.84		29.75± 9.47		14.67±5.18		25.26±7.08	
Additional work assignments								
No	64.24± 21.41	**0.037**	26.67±10.53	**0.046**	13.49± 5.18	**0.035**	24.09± 7.45	0.097
Yes	67.74± 20.01		28.30±9.68		14.37±5.10		25.07±7.13	
**Working environment**								
Poor	71.84± 20.44	**<0.001**	30.90±9.51	**<0.001**	15.34± 5.11	**<0.001**	25.60± 7.37	**0.002**
Good	61.38±19.59		24.72±9.65		12.83± 4.88		23.83± 7.10	

Note: numbers in bold represent significant at p<0.05.

#### Correlates of burnout

Table 3 shows the results of multiple stepwise linear regression analyses with MBI and subscales scores (EE, DP, DPA) as the dependent variables, while individual and environmental factors as independent variables. For the total MBI scale, four factors remained significant in backward regression: self-rated physical health (β = -6.68, p<0.001), working environment (β = -6.51, p<0.001), overwork (β = 4.13, p = 0.008), and satisfaction with income (β = -3.31, p = 0.007). For the cross-comparisons of the correlates of the three subscales, self-rated physical health and working environment remained consistently significant across EE, DP, and DPA, while satisfaction with income remained significant across EE and DP. In addition, overwork remained significant for EE only, while age remained significant for DPA only.

**Table 3:** Stepwise regression analysis of the influencing factors of burnout and its three dimensions (n = 623).

Dependent variable	Independent variable(step)	Standardized coefficient (β)	P value	95% CI
Total burnout	Self-rated health	-6.68	<0.001	(-8.98, -4.38)
	Working environment	-6.51	<0.001	(-9.88, -3.14)
	Overwork	4.13	0.008	(1.07, 7.19)
	Satisfaction with income	-3.31	0.007	(-5.68, -0.93)
Emotional exhaustion (EE)	Self-rated health	-3.97	<0.001	(-5.03, -2.90)
	Working environment	-3.81	<0.001	(-5.38, -2.24)
	Overwork	2.69	<0.001	(1.27, 4.12)
	Satisfaction with income	-1.95	0.001	(-3.06, -0.85)
Depersonalization (DP)	Self-rated health	-1.6	<0.001	(-2.17, -1.02)
	Working environment	-1.52	<0.001	(-2.37, -0.67)
	Satisfaction with income	-1.02	0.001	(-1.62, -0.42)
Decreased personal achievement (DPA)	Working environment	-1.52	0.009	(-2.66, -0.37)
	Self-rated health	-1.4	0.001	(-2.24, -0.56)
	Age	0.93	0.041	(0.04, 1.83)

## Discussion

### Summary of findings

To our knowledge, this is the first study to comprehensively examine the level of burnout (including the total MBI scale and its three subscales) and explore its individual and environmental correlates among a provincial-level sample of nurses from the hepatological surgery department. Our results showed a generally high level of burnout among hepatological surgery nurses with a high score in the total MBI scale and its three subscales. The prevalence of high burnout varied across the three subscales, indicating different levels of burnout by dimensions. Better self-rated health, a better working environment, no overwork, and satisfaction with income were associated with a lower risk of general burnout. For the three dimensions of burnout, self-rated physical health and the working environment remained consistently significant correlates across EE, DP, and DPA, while each subscale also has its additional unique correlates.

### Levels of burnout

Our study showed a significantly high level of burnout in each dimension among hepatological surgery nurses in Hunan Province. These scores were worse than the reported 23.95 ± 11.11 for EE, 7.90 ± 6.5 for DP and 27.51 ± 10.96 for PA in Wang et al [42]’s study among general nurses in Tianjin Province of China. But these scores were generally comparable to the reported 30 (26–34) for EE, 11 (8–14) for DP, and 23 (20–26) for PA in Meng et al [25]’s study among nurse anesthetists in Shandong Province. The prevalence of high burnout for EE (52.81%), DP (83.79%), and DPA (90.37%) was much higher than the reported 11.34% for EE, 7.4% for DP, and 64.02% for DPA in Guo et al [29]’s recent study among Hemodialysis nurses in 31 provinces in mainland China. The prevalence of high burnout in our study was also much higher than the reported 28% in the US [43], and the reported 11.23% by a recent systematic review and meta-analysis among global nurses across multiple specialties in 49 countries [9]. In general, our results showed an alarmingly higher level of burnout among hepatological surgery nurses in Hunan Province than most of the other studies conducted in other areas of China or other countries. Several reasons may explain the high burnout rate in our study. First, most patients cannot eat after hepatological surgery and rely on nasogastric tubes for nutrition intake, which may increase both the workload and difficulty level of nursing. Second, patients with hepatological surgery are at high risk of post-surgery adverse events such as drain falling off, and it requires expertise, experience, time, and patience for nurses to care for such patients. Third, most patients in hepatological surgery department were under serious condition such as liver cancer, the poor prognosis and low survival may add additional emotional burden to the nurses who are in direct contact with the patients. Furthermore, under the global pandemic of COVID-19, the strict safety requirement in the hospital adds extra burden to the nurses who are already overwhelmed by their intensive routine nursing work [44]. Additionally, as frontline health care providers, nurses are at high risk of getting infected with Covid-19 and infecting others, which further aggravate their psychological distress [44]. All these factors may contribute to the high burnout in nurses of hepatological surgery department in our study. This calls for high importance to be attached to the phenomenon of burnout and active measures to be taken to alleviate burnout among hepatological surgery nurses in Hunan Province.

Among the three dimensions of burnout, the DPA dimension showed the highest burnout prevalence, with over 90% of nurses reporting decreased personal achievement, a pattern consistent with most of the other studies in China [29]. Apart from systematic reasons such as the prevalent nursing staff shortage and high workloads among nurses all over the country, several specific work-related characteristics of the sample may also explain the nurses’ low level of personal achievement in the study. For instance, the majority of nurses were in contract employment, with the lowest professional and job titles, which all indicate low career development prospects and as a result low personal achievement from work. These findings have implications for future intervention programs to focus on improving nurses’ career development to improve their achievement and finally to reduce burnout. This may be realized through multiple career training programs to strengthen their competency as well as the expansion of various promotion opportunities to advance their professional development.

### Correlates of burnout

The study showed that self-rated physical health and working environment were the only two common correlates across all three dimensions of burnout: EE, DP, and PA. Compared to nurses with poor physical health, nurses with good physical health were at lower risk of burnout, reflected in lower levels of emotional exhaustion and depersonalization, and a higher level of personal achievement. This finding was consistent with previous findings showing poor physical health as a risk factor for burnout [25,32]. Nurses act as professional multi-tasking caregivers at hospitals and undertake many important duties to care for patients and support other health providers. Apart from direct patient work such as medication administration, post-surgery care, medical information and records updates, and routine monitoring of patients, nurses also shoulder the task of communication among patients, families, and other health providers to coordinate and implement treatment and care plans. The multiple high-intensity nursing duties, combined with irregular working hours and night shifts all require nurses to have good physical health to maintain their work. Nurses with better physical health are more competent to satisfy the diverse and high-demanding working requirements, and as such, are less likely to suffer from the emotional overextension and depletion resulting from work stress, to show negative attitudes towards patients, and to feel incompetent or dissatisfied with their work.

On the other hand, the negative association between physical health and burnout may reflect a bidirectional relationship between them, that is, not only does poor physical health leads to high burnout, but also high burnout leads to poor physical health. As previous evidence has robustly demonstrated the negative impact of burnout on physical health, including fatigue, headache, insomnia, appetite changes, chest pain, frequent colds, gastrointestinal distress, heart disease, and even mortality [13,14]. Our findings underline the importance of maintaining nurses’ physical health in combatting their job burnout, which may be realized through promoting healthy lifestyles such as healthy eating and regular exercise among nurses through education and propaganda. Our study also suggests for future longitudinal studies to explore the potential bidirectional relationship between physical health and burnout.

Consistent with the bulk of literature highlighting the essential role the working environment played in nurse burnout [12,19,21,29,33,34], our study also showed that a good working environment was strongly associated with a lower risk of burnout. To be specific, nurses with high levels of hospital and department support, harmonious colleague working relationship, and received high respect from patients had lower levels of emotional exhaustion and depersonalization, and a higher level of personal achievement. Substantial previous evidence has documented that the lack of support from leadership, lack of collaboration among colleagues, and lack of patient trust and respect were leading factors that contribute to nursing burnout [21,29]. Our findings add further support to the previous studies and suggest improvements in the working environment to reduce nurse burnout, which has already been may significantly decrease nurse burnout, which may significantly minimize the negative impacts of nurse burnout on patient outcomes as well as improve patient satisfaction [19,34,45,46].

Apart from the two common correlates across all three dimensions of burnout, our study also showed each dimension has its unique additional correlates. For instance, EE was associated with overwork and satisfaction with income, DP was associated with satisfaction with income, and DPA was associated with age. The positive associations between satisfaction with income and low levels of EE and DP were in alignment with previous studies showing higher income as a protective factor for job burnout [25,28]. This finding suggests raise in income may be beneficial in improving nurses’ burnout in terms of EE and DP. Having to work overtime indicates a higher level of job stress, which has been consistently shown to be contributing factor leading to emotional distress [42]. This finding suggests that reducing workload and avoiding overwork can help decrease nurses’ emotional exhaustion. The higher level of burnout in terms of personal achievement among older nurses may reflect a lower professional development room with aging and suggest the need of providing more advanced education and training opportunities for older nurses to improve their achievement. In summary, these findings underscore the different correlates of various dimensions of burnout, which may require different targeted interventions.

### Limitations

This study has several limitations that should be considered when interpreting the results. First, the sample was limited to hepatological surgery nurses in Hunan Province; the results, therefore, may not extend to nurses in other specialties, or in other areas of China. Future studies may consider using nationally representative samples of nurses in various specialties to get a more comprehensive picture and facilitate better comparisons. Second, the cross-sectional study design makes it impossible to establish causal relationships between risk factors and burnout. Future longitudinal or interventional studies are highly recommended to robustly demonstrate causal relationships. Third, the evaluation of the working environment was based on a self-designed 4-item scale, which may not represent the psychosocial work environment fully. Future studies should consider including a broad assessment of psychosocial environment factors such as risky work tasks, work demands, work content (task clarity, unexpected responsibilities), decision latitude, decision-making authority.

## Conclusions

In this study on burnout among hepatological surgery nurses in Hunan Province, we found alarmingly high levels of burnout in terms of emotional exhaustion, depersonalization, and decreased personal achievement, which deserves public attention and calls for urgent interventions to relieve nurse burnout. Self-rated physical health and the working environment remained two common correlates across all three dimensions of burnout, highlighting the improvement of physical health and the working environment of nurses may be the most beneficial intervention measures to tackle various dimensions of burnout. Apart from the common correlates, each dimension of burnout has its unique additional correlates such as overwork, satisfaction with income, and age, implying the need for targeted interventions for each specific dimension of burnout.
